# P-356. Preliminary Experience using a Pharmacist-led Intervention to Support Injectable HIV Treatment for Women with Health-related Social Needs

**DOI:** 10.1093/ofid/ofaf695.574

**Published:** 2026-01-11

**Authors:** Jaimie P Meyer, Carolina R Price, DeShana Tracey, Ana Hernandez, Natalie Tucker, Bailey Thayer, Lydia A Barakat

**Affiliations:** Yale School of Medicine, New Haven, CT; Yale School of Medicine, New Haven, CT; Yale School of Medicine, New Haven, CT; Yale School of Medicine, New Haven, CT; Yale New Haven Hospital, New Haven, CT; Yale New Haven Hospital, New Haven, CT; Yale School of Medicine, New Haven, CT

## Abstract

**Background:**

Women with health-related social needs (HRSN) are often excluded from consideration for CAB/RPV LA because of concerns that HRSN may interfere with injection visit adherence. In Project Tara, an ongoing single-arm pilot study, we use an enhanced pharmacist-led collaborative drug therapy management model (CDTM+) for CAB/RPV LA to reach women with HRSN and are evaluating patient-reported outcomes and CAB/RPV LA initiation.

**Methods:**

Participants are recruited from the largest Ryan White-funded HIV clinic in New Haven, CT and screened for these criteria: 1) female; 2) HIV diagnosis; 3) on oral ART and virally suppressed for ≥3 months; and 4) report ≥1 current HRSN. Participants complete study interviews over 6 months and meet with a clinic-based pharmacist for CAB/RPV LA education, clinical eligibility evaluation, and initiation if interested. We conducted an interim descriptive analyses of baseline characteristics and intervention engagement and outcomes.

**Results:**

After screening (n=64), we have enrolled 25 women with HIV and HRSN, who are generally older (mean age=60.6y [SD 7.4]) and racially diverse (Black (72%); white (24%); Hispanic/Latine (8%)). Participants had HIV diagnosed a mean of 28.4 years (SD 9.8) prior, with mean baseline CD4 count= 651 cells/µL (SD 336); nearly all (76%) have viral loads < 20 copies/mL. Participants have multiple HRSN, including: housing instability or poor living conditions (84%), food insecurity (60%), lifetime criminal legal system involvement (88%), moderate to high-risk substance use (72%) primarily cocaine, alcohol misuse (16%), and depression (24%). To date, nearly all (88%) completed a pharmacist visit, resulting in 4 expressing interest in starting CAB/RPV LA, and 2 having received injections. Others declined CAB/RPV LA (n=9), deferred the decision (n=9), or were clinically ineligible (n=4) due to NNRTI resistance or chronic hepatitis B.

**Conclusion:**

Although women with HRSN are often not considered for or are unable to access CAB/RPV LA, we found that CDTM+ is resulting in new initiations of CAB/RPV LA through facilitated access and support. Empowering women with HRSN with knowledge about and access to long-acting injectable ART is important for achieving health equity.

**Disclosures:**

Jaimie P. Meyer, MD, MS, FACP, ViiV Healthcare: Grant/Research Support

